# Recombinant Expression, Purification, and Functional Characterisation of Connective Tissue Growth Factor and Nephroblastoma-Overexpressed Protein

**DOI:** 10.1371/journal.pone.0016000

**Published:** 2010-12-30

**Authors:** Wilhelm Bohr, Michael Kupper, Kurt Hoffmann, Ralf Weiskirchen

**Affiliations:** 1 Institute of Clinical Chemistry and Pathobiochemistry, RWTH-University Hospital Aachen, Aachen, Germany; 2 Institute for Molecular Biotechnology, RWTH-Aachen University, Aachen, Germany; University of South Florida College of Medicine, United States of America

## Abstract

The CCN family of proteins, especially its prominent member, the Connective tissue growth factor (CTGF/CCN2) has been identified as a possible biomarker for the diagnosis of fibrotic diseases. As a downstream mediator of TGF-β1 signalling, it is involved in tissue scarring, stimulates interstitial deposition of extracellular matrix proteins, and promotes proliferation of several cell types. Another member of this family, the Nephroblastoma-Overexpressed protein (NOV/CCN3), has growth-inhibiting properties. First reports further suggest that these two CCN family members act opposite to each other in regulating extracellular matrix protein expression and reciprocally influence their own expression when over-expressed. We have established stable HEK and Flp-In-293 clones as productive sources for recombinant human CCN2/CTGF. In addition, we generated an adenoviral vector for recombinant expression of rat NOV and established protocols to purify large quantities of these CCN proteins. The identity of purified human CCN2/CTGF and rat CCN3/NOV was proven by In-gel digest followed by ESI-TOF/MS mass spectrometry. The biological activity of purified proteins was demonstrated using a Smad3-sensitive reporter gene and BrdU proliferation assay in permanent cell line EA•hy 926 cells. We further demonstrate for the first time that both recombinant CCN proteins are N-glycosylated.

## Introduction

Fibrosis is characterised by the deposition of the extracellular matrix (ECM) proteins including collagens, structural glycoproteins, sulphated proteoglycans and hyaluronan into the subendothelial space of Disse [1]. The biomechanical and biochemical rebuilding of the tissue is induced by several important fibrogenic cytokines including transforming growth factor (TGF-β, platelet derived growth factor-B and -D (PDGF-BB, PDGF-DD), insulin-like growth factor-1 (IGF-1) and endothelin-1 (ET-1) [2]. TGF-β assists the wound repair and ECM deposition through intracellular Smad proteins that transfer signals from the cell membrane to the nucleus [3] and is of fundamental importance for the development of excess fibrous connective tissue [4]. In this process, the Connective Tissue Growth Factor (CCN2/CTGF) appears as the downstream modulator of TGF-βsignalling. In liver for example, CCN2/CTGF expression is *in vitro* triggered in hepatocytes, fibroblasts and other cell types after stimulation with TGF-β [5], [6]. *In vivo* it could be shown, that the expression of CCN2/CTGF was persistent after TGF-β stimulation. It acts as a cofactor with TGF-β to induce hepatic fibrogenesis but is not considered to be a potent fibrogenic agent on its own [7]. However, one of its important functions is to stimulate the expression of ECM and proliferation of connective tissue cells to close wounds inside of internal organ and skin tissue [8]–[10].

CCN2/CTGF is one of the six members of the CCN protein family. The name of the family is an acronym of the first three identified members, namely CYR61 (CCN1), CTGF (CCN2) and the Nephroblastoma-overexpressed gene (NOV, CCN3). The other members are WISP1 (ELM1, CCN4), WISP2 (COP-1, CCN5) and WISP3 (CCN6). The different CCN proteins have a similar predicted secondary structure consisting of 5 individual modular domains that includes an insulin-like growth factor binding domain, a von Willebrand factor type C repeat, a thrombospondin type 1 repeat and a carboxyl-terminal cysteine knot [11], [12]. Moreover, the CCN proteins show a high sequence identity between species with a highly conserved intramolecular network of cysteine residues [13]. They own a variety of biological functions that is strongly dependent on their cellular microenvironment [14] and fine-tuned by the activity and interplay of the individual structural domains [15].

CCN2/CTGF is a 38-kDa protein was originally identified as a growth factor secreted by human vascular endothelial cells [16] and defined as the chondrocyte-specific gene product [17], [18]. It is over-expressed in many fibrotic disorders including scleroderma, kidney and liver fibrosis [19]–[21], [12] and constitutive expression of CCN2/CTGF in tissues is considered to indicative for pathological lesions [5]. CCN2/CTGF promotes endothelial proliferation, migration, survival and adhesion in angiogenesis [22]. Furthermore, CCN2/CTGF acts as an immediate early gene that are induced by serum or growth factors and believed to play important roles in the control of cell proliferation [9]. The TGF-β dependency of CCN2/CTGF is mainly controlled by sequence elements within the promoter that include a Smad-binding site and a defined TGF-β response element [20], [23].

Another member of the CCN family is the CCN3/NOV protein that under certain conditions counteracts CCN2/CTGF functions [24], [25]. CCN3/NOV was first identified in Myeloblastosis-associated virus-1-induced avian nephroblastomas [26], [27]. It is over-expressed in a broad range of tumors including Wilm's tumor and is further associated with neovascularisation and vascular injuries in which it promotes pro-angiogenic activities through integrin receptors in vascular endothelial cells [28], [29]. Some other studies have shown that CCN3/NOV inhibits glioma cell and PDGF-dependent glomerular cell proliferation [30], [31].

However, the molecular understanding of CCN2/CTGF and CCN3/NOV is still at the beginning and several key questions are still unanswered. In particular, data is missing that explains the regulatory network in which CCN2/CTGF promotes TGF-β action that is necessary to maximally induce certain genes including collagen type I and α-smooth muscle actin. Moreover, the mechanisms that are required for the reported Yin/Yang regulation of CCN2/CTGF and CCN3/NOV are still an enigma. Presently, the performance of studies addressing these mechanistic issues are somewhat limited by the lack of larger quantities of biological active of respective CCN proteins.

In the present study, we aimed to develop devices that allow the production of biological active recombinant CCN2/CTGF and CCN3/NOV. We report about the establishment of transgenic cell lines that over-express human CCN2/CTGF and an adenoviral expression vector for production of rat CCN3/NOV. We have established protocols for purification of these two CCN, and biochemically and biologically characterised the recombinant proteins by mass spectrometry, tryptic in-gel-digests, and proliferation and gene reporter assays in EA•hy 926 cells. Moreover, we demonstrate for the first time that both CCN2/CTGF and CCN3/NOV are N-glycosylated and that the predicted Yin/Yang activity both CCN proteins can be demonstrated in EA•hy 926 cells.

## Materials and Methods

### Reagents

Recombinant TGF-β1, PDGF and the goat anti-mouse CCN3/NOV polyclonal antibody AF1976 were obtained from R&D Systems (Wiesbaden-Nordenstadt, Germany). The goat anti-human CCN2/CTGF polyclonal antibody L-20 (sc-14939) was obtained from Santa Cruz Biotechnology (Santa Cruz, CA). 1-Step BrdU ELISA was obtained from Roche Biosciences (Mannheim, Germany). The (CAGA)_12_-MLP-Luc vector [32] was kindly provided by Peter ten Dijke (Department of Molecular Cell Biology, Leiden University Medical Center). All chromatography columns (HiTrap™ Heparin HP, HiLoad 16/60 Superdex) were obtained from GE Healthcare Life Sciences (GE, München, Germany).

### Cell lines and culture

Flp-In-293 cells were obtained from Invitrogen (Darmstadt, Germany). Parental HEK cells and COS-7 were obtained from the ATCC (Manassas, VA). EA•hy 926 cells [33] were kindly provided by Dr. Cora-Jean Edgell (Department of Pathology and Laboratory Medicine, Carolina Cardiovascular Biology Center, Chapel Hill, NC). All cell lines were cultured in Dulbecco's Modified Eagle's medium (DMEM) (BioWhittaker, Verviers, Belgium) supplemented with streptomycin (100 µg/ml), penicillin (100 IU/ml), L-glutamine (4 mM) and 10% fetal calf serum (FCS, v/v) at 37°C in a humidified atmosphere containing 5% (v/v) CO_2_. All cell culture supplements were obtained from PAA Laboratories (Pasching, Austria). The HEK or Flp-In-293 clones expressing recombinant human CCN2/CTGF (rhCTGF) were cultured in the same medium containing additional 150 µg/ml Hygromycin B (Roth, Karlsruhe, Germany). All cells were cultivated until they reached 70–80% confluence. For subcultivation, they were washed 3 times with 1× phosphate-buffered saline (PBS, pH 7.4) and incubated for 15 min with Accutase™ (PAA), and plated on new cell culture dishes. The EA•hy 926 cells were sub-cultivated by use of 2× trypsin (PAA).

### Transfection experiments

All transfections were done with the FuGENE 6 transfection reagent (Roche) according to the manufacturer's instructions. Briefly, HEK cells were seeded at 2×10^5^ cells per 6-well plate (Sarstedt, Nürnbrecht, Germany) and incubated overnight in culture medium. The medium was renewed and cells were cultured for another 2 h period prior transfection. Sixteen hours later, the medium was changed and clones were selected with indicated antibiotics.

### Stable expression of human CCN2/CTGF in HEK cells and in the Flp-In-293 systems

The full length human CCN2/CTGF cDNA sequence was amplified from expression clone pCEP4-hCTGF [34] that was kindly provided by W. Sebald (Department of Physiological Chemistry II, Theodor-Boveri Institute for Life Sciences (Biocenter), University of Wuerzburg, Germany) using primers hCTGFfor (5′-GAA TTC ATG ACC GCC GCC AGT ATG GG-3′) and hCTGFrev(5′-CTC GAG TGC CAT GTC TCC GTA CAT CT-3′). The amplicon was cloned into the pGEM™-Easy cloning vector (Promega, Mannheim, Germany). Subsequently, the cDNA was released by the *Not*I endonuclease and subcloned into the multiple cloning site of vector pcDNA5/FRT/TO (Invitrogen) designed for use with the Flp-In-293 system that allows production of stable cell clones expressing the gene/protein of interest. The correct orientation within vector pcDNA5/FRT/TO-hCTGF was verified by sequencing. This vector system contains a Hygromycin B resistance gene cassette allowing selection and maintenance of transgenic clones. For generation of stable expression clones, cells (HEK293A or Flp-In-293) were seeded on 6-well plates and transfected with pcDNA5/FRT/TO-hCTGF alone (HEK293A) or pcDNA5/FRT/TO-hCTGF and pOG44 (Invitrogen) (Flp-In-293) containing the transferase/integrase gene. Transfected cells were selected with increasing concentrations of Hygromycin B ranging from 50–400 µg/ml and dying of untransfected cells was visualized by light microscopy. The ideal concentration was determined to 150 to 200 µg/ml of Hygromycin B for both cell lines. Individual cell clones were established by limiting dilution (0.5 cells/well in a 96-well plate) and numbered consecutively (HEK clones: 1/1, 1/2, 1/3 etc.; Flp-In-293 clones: WB1, WB2, WB3 etc.). Expression and secretion of recombinant CCN2/CTGF was analyzed in ELISA [35] and Western blot using the CCN2/CTGF-specific antibody sc-14939 (Santa Cruz). Stable cell clones were frozen at −80°C using standard cryoconservation protocols. Five of 120 initial cell clones were further expanded for production of recombinant CCN2/CTGF. The expression of recombinant CCN2/CTGF in each of both systems (HEK, Flp-In-293) was documented for over 100 passages.

### Immunocytochemistry

HEK and Flp-In-293 cells were seeded on poly-L-lysine-coated glass cover slips that were prepared following a standard protocol. On the next day, the medium was renewed and cells were incubated for further 24 hr. Then the cells were washed in PBS, fixed in 4% (v/v) paraformaldehyde in 1× PBS (pH 7.4) for 10 min, and permealized for 2 min on ice in 0.1% (w/v) sodium citrate and 0.1% (v/v) Triton X. Cells were blocked for 10 min at RT in Biotin Blocking Reagent (X0590) obtained from DAKO (Hamburg, Germany) following manufacturer's instructions. Unspecific binding sites were further blocked in 50% (v/v) FCS, 1% BSA (w/v), and 0.1% (v/v) fish gelatine in PBS (pH 7.4) for 1 h. Finally, the cells were incubated overnight at 4°C in a 1∶300 dilution of the anti-human CCN2/CTGF antibody (sc-14939) in PBS containing 1% (w/v) BSA. Cells were washed and incubated with an anti-goat-biotinylated antibody (1∶300, DAKO) for 1 h, and further with a Streptavidin/FITC-conjugate for 30 min at RT. The nuclei were stained with DAPI. As a control, the cells were incubated with an unspecific immunoglobulin.

### Western blot analysis

The cells were washed three times in PBS and lysed in 250 µl RIPA buffer (50 mM Tris/HCl (pH 7.4), 1% (v/v) NP-40, 0.25% (w/v) sodium deochycholate, 150 mM NaCl, 0.1% (w/v) SDS, 1 mM EDTA, 1 mM PMSF, 1 mM Na_3_VO_4_, and 1 mM NaF) per well of the 6-well plate. The protein concentration was measured using the Micro BCA protein assay (Pierce Thermo Scientific, Bonn, Germany). Proteins were separated in Bis/Tris 4–12% SDS-PAGE (Invitrogen) in MES buffer and transferred to nitrocellulose membranes (Schleicher & Schüll, Dassel, Germany). For documentation of equal protein loading, the membranes were stained with Ponceau S and blocked for 30 min in 5% non-fat dried milk dissolved in TBST containing 10 mM Tris (pH 8.0), 150 mM NaCl, and 0.1% (v/v) Tween-20. The membranes were incubated overnight with a goat-anti-CCN2/CTGF (sc-14939) at 4°C under permanent shaking. The membranes were rinsed three times in TBST and incubated with a monoclonal donkey anti-goat-HRP conjugate (sc-2056, Santa Cruz) at RT for 1 h and signal detection was conducted by use of the SuperSignal West Pico Chemiluminescent Substrate (Pierce).

### Generation of adenoviral expression vector AdEasy-CMV-rNOV

To subclone the coding region of rat CCN3/NOV, a PCR was performed using plasmid clone IRAKp961P24175Q (ImaGENE, Berlin, Germany) as a template and primers 5′-(TTG TAG AAT TCA GCA GGC AGA ACA TG)-3′ and 5′-(TTA CCG GTA CAT TTC TCC TCT GCT)-3. The amplicon was first cloned into pGEM-T-Easy. Subsequently, the NOV containing sequence was released by *Eco*RI/*Age*I and cloned into respective sites of vector pcDNA3.1/V5-HisA. Thereafter, the CCN3/NOV expression cassette was cut out by digest with *Eco*RI and *Pme*I, blunted by Klenow, and cloned into vector adenoviral shuttle vector pShuttle-CMV (Stratagene, Agilent Technologies, Waldbronn, Germany). For generation of a recombinant adenoviral plasmid, the resulting vector pShuttle-CMV-rNOV was linearized by *Pme*I digest and cotransfected with adenoviral backbone vector pAdEasy-1 (Stratagene) into electroporation competent BJ5183 bacteria. A recombinant vector pAdEasy-1-CMV-rNOV was isolated, characterized by restriction digest and sequencing, and transfected into 293A cells to prepare adenoviral particles using standard transfection, amplification and purification techniques.

### Purification of recombinant CCN2/CTGF

For production of recombinant human CTGF, transgenic cells were seeded at 2×10^6^ cells/culture dish and cultivated in growth medium for 24 h. The medium was replaced one day later with medium without FCS and expression/secretion of CCN2/CTGF was allowed for 48 h. For purification of CCN2/CTGF, we followed a two-step protocol. In the first step (heparin affinity chromatography), we took advantage of the ability of CCN2/CTGF to bind to heparin. The enriched growth medium was centrifuged for 20 min at 5000×g and supernatant was filtered (0.22 µm). Then the growth medium was equilibrated with 1/10 VT of 100 mM Tris/HCl (pH 7.0) and subjected with a syringe to a HiTrap™ HP Heparin column (5 ml) that was equilibrated with 25 ml of 10 mM Tris/HCl (pH 7.0). The column was then washed with 25 ml 10 mM Tris/HCl (pH 7.0) and the recombinant protein was stepwise eluted from the heparin matrix with 10 mM Tris/HCl (pH 7.0) containing increasing concentrations of NaCl (25 ml 0.15 M NaCl; 15 ml 0.4 M NaCl; and 10 ml each of 0.4 M, 0.6 M, 0.8 M, 1.0 M, 1.2 M and 4 M NaCl). The purest CCN2/CTGF fraction was eluted at 0.7 M NaCl. The content of each fraction was tested by Western blot or ELISA. CTGF containing fractions were pooled and subjected to a second cycle of heparin affinity chromatography. Then the 0.7 M NaCl fraction of the second Heparin affinity chromatography was applied to a Superdex Sepharose 16/60 size-exclusion chromatography that was run on a FPLC chromatographic system from GE Biosciences equipped with a P-900 photometer. Fractions of 3 ml were collected and tested for the content of recombinant CCN2/CTGF by ELISA or Western Blot. The purity of protein solution was evaluated by SDS-PAGE and Coomassie Brilliant Blue staining. All buffers and solutions used during the purification of rhCTGF was filtered (0.22 µm) filtrated and kept at 4°C.

### Purification of recombinant CCN3/NOV

The purification of recombinant rat NOV was performed from pooled supernatants of COS-7 cells that were infected with the adenoviral vector Ad-CMV-NOV and later harbored in DMEM without FCS 48 h. After centrifugation (20 min, 5000×g) and filtration (0.22 µm), the equilibrated supernatant (10 mM Tris, pH 7.0) was subjected to heparin affinity chromatography. After washing (25 ml of 10 mM Tris/HCl pH 7.0 and 25 ml of 10 mM Tris/HCl pH 7.0/0.15 M NaCl), recombinant NOV was eluted with 0.8 M NaCl in 10 mM Tris/HCl (pH 7.0). In a second step, NOV containing fractions were pooled and further purified on a HiLoad 16/60 Superdex™ column with 10 mM Tris/HCl (pH 7.0) as running buffer.

### CCN2/CTGF ELISA

The ELISA for quantification of human CCN2/CTGF was described before [35]. Briefly, the antibodies (rabbit-anti-human CCN2/CTGF (sc-25440) for plate coating, goat-anti human CCN2/CTGF (sc-14939) for detection, and donkey anti-goat IgG-HRP (sc-2056) for visualisation) were all obtained from Santa Cruz. The microtiterplates were first coated overnight at 4°C with a 1∶1000 dilution of antibody sc-25440 in coating buffer [50 mM natrium carbonate (pH 9.6): 1.59 g/l Na_2_CO_3_ and 2.93 g/l NaHCO_3_]. The plates were then rinsed three times with PBST (PBS with 0.05% Tween-20) and supernatants were mixed with an equal volume of PBST and dispensed into the wells and incubated at RT under shaking for 2 h. The probes removed, wells rinsed with PBST and the second antibody against CCN2/CTGF (sc-14939) dispersed in a 1∶1000 dilution into every well and incubated for 2 h at RT. After a further washing, the anti-goat IgG-HRP conjugate was dispersed in a 1∶15000 dilution for 1 h. The plates were washed three times and developed by addition of the 3,3′,5,5-tetramethyl benzidine (TMB) substrate at RT for 10 min. The reaction was stopped in 2 M H_2_SO_4_ and the absorbance at 450_ nm_ was determined in a Wallac Victor 1420 Multilabel counter. The concentration was measured in duplicate.

### 2-D PAGE and mass-spectrometric analysis

Immobilized pH gradient strips (ReadyStrip™ IPG Strip, pH range 3–10, #163-2000, Bio-Rad Laboratories, Philadelphia, PA) were re-hydrated in the PROTEAN® IEF focusing tray. The protein sample was re-suspended in 125 µl of re-hydration buffer (9 M Urea, 4% CHAPS, 0.4% Ampholyte, 65 mM DTT and Bromphenol Blue) and applied as a line along the edge of channel in the PROTEAN® IEF focusing tray and covered with the immobilized pH gradient (IPG) strips (7 cm length). Before starting the active re-hydration, the IPG strip was passively re-hydrated for one hour until the liquid was absorbed by the gel bed. The IPG strips with the re-hydration buffer/protein solution were covered with mineral oil to prevent the evaporation during active re-hydration and entrance of the proteins into the gel of the IPG strip overnight. Wet electrode wicks were inserted between the IPG strips and the electrodes. The active re-hydration of the protein species on the IPG strips was done in the PROTEAN® IEF Cell overnight (16 hr): 50 V, 20°C. The focussing program was performed in the PROTEAN® IEF Cell (Bio-Rad). The first step of focusing was done rapidly linear at 500 V for 250 Vh, the second step was done from rapidly linear at 1000 V for 500 Vh. The third step was done rapidly linear at 8000 V for 6500 Vh. The program ended at constant 500 V.

Before running the second dimension, the IPG strips were equilibrated in SDS-containing buffers. The first equilibration buffer containing DTT reduces sulfhydryl groups and the second equilibration buffer containing iodoacetamide alkylates the reduced sulfhydryl groups. Briefly, the strips were shortly washed with Milli-Q water and soaked for 15 min in a buffer containing 6 M Urea, 30% (v/v) glycerol, 2% (w/v) SDS, 0.065 M DTT, and 0.05 M Tris (pH 8.8) on an orbital shaker. Then, the strips were shortly washed in Milli-Q water and equilibrated for 15 min in a buffer containing 6 M urea, 30% (v/v) glycerol, 2% (w/v) SDS, 0.135 M iodoacetamide, and 0.05 M Tris (pH 8.8) on the orbital shaker. The equilibrated IPG strips were placed on the top of the 12% SDS-PAGE that was run in Tris/glycine buffer (248 mM Tris, 192 mM glycine, 1% (w/v) SDS, pH 8.3) for 20 min at 120 V to force the immobilized protein spots from the strip. Subsequently, the separation of the protein spots was performed for 40 min at 180 V. The final gels were analysed by colloid Coomassie staining, Western blot and in gel digestion was realized prior to ESI-MS/MS analysis.

### ESI/TOF-MS/MS

The identity of CCN3/NOV and CCN2/CTGF was verified by MS/MS-identification of individual fragments obtained by tryptic digestion. The polyacrylamide gel was rinsed two times in Milli-Q water to remove excess SDS from the gel, reducing strong background during sensitive Coomassie blue staining [36]. Then the gels were stained overnight in 5% (w/v) aluminium sulphate hexadecahydrate (06421; FLUKA, Sigma-Aldrich, Munich, Germany), 10% (v/v) ethanol, 0.02% (w/v) Coomassie Brilliant Blue (9598.1, CBB-G250, Roth) and 2% (v/v) of 85% o-phosphoric acid (79620, FLUKA). Thereafter, the gels were rinsed 2 times in Milli-Q water and bands of interest were excised with a clean scalpel avoiding background gel volume around of the band/spot. The excised bands were chopped into small cubes and transferred into a 1.5 ml microcentrifuge tubes. The gel cubes were washed with 150 µl of Milli-Q water and spinned down with maximum speed in a table centrifuge. The supernatant was replaced by 4 volumes of acetonitrile per volume of gel pieces and the gel pieces were dried for 15 min. After spinning in a microcentrifuge, the acetonitrile was removed and replaced by 10 mM DTT in 0.1 M NH_4_HCO_3_. After incubation (30 min, 37°C) the gel pieces were collected by centrifugation and the supernatant replaced with acetonitrile for 15 min. The gel pieces were spinned down again and the supernatant replaced with 55 mM iodoacetamide in 0.1 M (NH_4_)HCO_3_ and further incubated in the dark at RT for 20 min. The solution was then replaced and gel pieces soaked for 15 min in 200 µl 0.1 M (NH_4_)HCO_3_ to remove traces of iodoacetamide. The supernatant was removed after spinning and the gel pieces dried in the vacuum centrifuge. The gel pieces were re-hydrated in digesting buffer containing 50 mM NH_4_HCO_3_, 5 mM CaCl_2_ and 12.5 ng/ml trypsin at 4°C for 45 min, and incubated overnight at 37°C. 15 µl of 25 mM NH_4_HCO_3_ was added and gel pieces were centrifuged for 15 min. Two volumes of acetonitrile were added, and probes incubated at 37°C for 15 min with shaking, and the supernatants collected after centrifugation. 50 µl of 5% (v/v) formic acid was added and the suspension mixed for 15 min at 37°C. The extracts were pulled down and dried in a vacuum centrifuge. Identification of peptides was performed using a nanoHPLC (Dionex, Sunnyvale, CA) coupled to an ESI-MS/MS mass spectrometer (Micromas electrospray Q-TOF-2, Waters Corporation, Milford, MA). Protein identification was carried out using the search engine MASCOT (Matrix Science, Boston, MA).

### MALDI-TOF mass spectrometry

Desalting of peptides was done by using the ZipTipC_μ-C8_ devices according to the manufacturer's instruction (Millipore Corporation, Billerica, MA). The dried protein samples were resuspended in 10 µl of 5% (v/v) formic acid in HPLC pure water. The ZipTipC_μ-C8_ was pre-wetted in 25% (v/v) of mixture of 5% (v/v) formic acid in HPLC pure water and 75% (v/v) acetonitrile and equilibrated with 5 volumes of 5% (v/v) formic acid in HPLC pure water. The proteins were applied on the ZipTipC_μ-C8_ in 10 aspiration and dispensing cycles. Then, the surface of the column of the ZipTipC_μ-C18_ was washed with 5 volumes of 5% (v/v) formic acid in HPLC pure water and the proteins were eluted into a clean vial by dispensing of 2–4 µl of elution solution containing 25% (v/v) of a 5% (v/v) aqueous formic acid solution in HPLC pure water and 75% (v/v) acetonitrile. The solution was aspirated and dispensed through the ZipTipC_μ-C8_ at least 10 times. Subsequently, 1 µl of the eluted protein was dried on the 96-well MALDI target plate (Gilson, Middleton, USA) and 1 µl of the Matrix solution containing sinapinic acid in 30–50% of acetonitrile, 0.1% trifluoracetic acid and HPLC pure water was pipetted on the dried protein drop. The mass of recombinant CTGF and NOV were determined with the MALDI-TOF/TOF mass spectrometer method (ultrafleXtreme MALDI-TOF/TOF mass spectrometer, Bruker Daltonics, Bremen, Germany). The protein standard II (Bruker) that contains three standard proteins (Trypsinogen, Protein A, Bovine Serum Albumin) with average m/z 23982, 44613, and ∼66500 was used for testing and calibration of the MALDI mass spectrometer.

### Deglycosylation of CCN2/CTGF and CCN3/NOV

The purified CCN proteins were deglycosylated according to the manufacturer's instructions (NEB, Ipswich, MA). For deglycosylation with the Endo-N-acetyl-β-glucosaminidase H (Endo H), 20 µg of recombinant CCN2/CTGF or CCN3/NOV were mixed with 1/10^th^ volume of 10× denaturating buffer (5% (w/v) SDS, 0.4 M DTT) and heated for 10 min at 100°C. Then, the solution was mixed with 1/10^th^ volume of the 10× reaction buffer (0.5 M sodium citrate, pH 5.5) and protein deglycosylation was performed with 1000 IU of Endo H for 1 h at 37°C. For deglycosylation with Peptid-N-(N-acetyl-β-glucosaminyl)-asparaginamidase (PNGase F), 20 µg of each protein was denaturated in 1× denaturation buffer at 100°C for 10 min. Then 1/10^th^ volume of each 10×G7 reaction buffer (0.5 M sodium phosphate, pH 7.5) and 10% (v/v) NP-40 was added and deglycosylation was performed by adding of 1000 IU of PNGase F for 1 h at 37°C. For glycosylation studies the following antibodies/conjugates were used: goat-anti-mouse CCN3/NOV (#AF1976, R&D Systems); goat-anti-human CCN2/CTGF (sc-14939) obtained from Santa Cruz; rabbit-anti-mouse PDGF type β receptor (#PC17) obtained from Oncogene (San Diego, CA); Con A-HRP (L6397) obtained from Sigma-Aldrich (Munich, Germany). Prediction of N-glycosylation sites was done using the NetNGlyc tool from the Expert Protein Analysis System (ExPASy) Proteomic Server at http://expasy.org/.

### Biological activity of purified CCN proteins

(i) Cell proliferation. The biological activity of purified recombinant CCN2/CTGF was tested om EA•hy 296 cells. Therefore, 3×10^4^ cells were seeded in the wells of a 12-well plate and cultured overnight in growth medium. The next day, the medium was changed to starvation medium (0% FCS, 1 mg/ml BSA) and cells were serum-starved for 16 hr. Thereafter, the medium was renewed, different amounts of recombinant CCN2/CTGF (100 ng to 1.5 µg/ml) added, and cells incubated for a further 24 hr period. Then the medium was replaced against labeling medium (DMEM, 1 mg/ml BSA, and 0.01 mM 5-bromo-2′-deoxyuridine) and cells incubated for a further 24 hr period. Finally, the incorporation of BrdU was quantified with the 1-Step™ BrdU ELISA (Roche) according to the manufacturer's instructions. The absorption was measured in a Wallac Victor 1420 Multilabel counter. (ii) Luciferase assay. EA•hy 926 cells were seeded with 1×10^5^ cells/well in 6-well plates and incubated overnight. The next day the medium was renewed and transfected with 1 µg of the (CAGA)_12_-MLP-Luc vector using 3 µl of the TransIT-LT1 transfection reagent (Mirus, VWR International, Darmstadt, Germany) after another 2-hr incubation time. The next day, the serum content of the medium was reduced to 0.5% FCS and cells starved for 16 hr. Thereafter, cells were stimulated in medium (0.2% FCS) with indicated concentrations of cytokines for 24 hr. Finally, cells were lysed in 1× luciferase lysis buffer (Promega), LAR substrate added, and the luciferase activity measured in a Wallac Multilabel counter. The luciferase activity given in light counts per second was normalized against the protein concentration in each sample and given as relative light units (RLU).

## Results

### Purification of recombinant human CCN2/CTGF and recombinant rat CCN3/NOV

**Figure 1:**
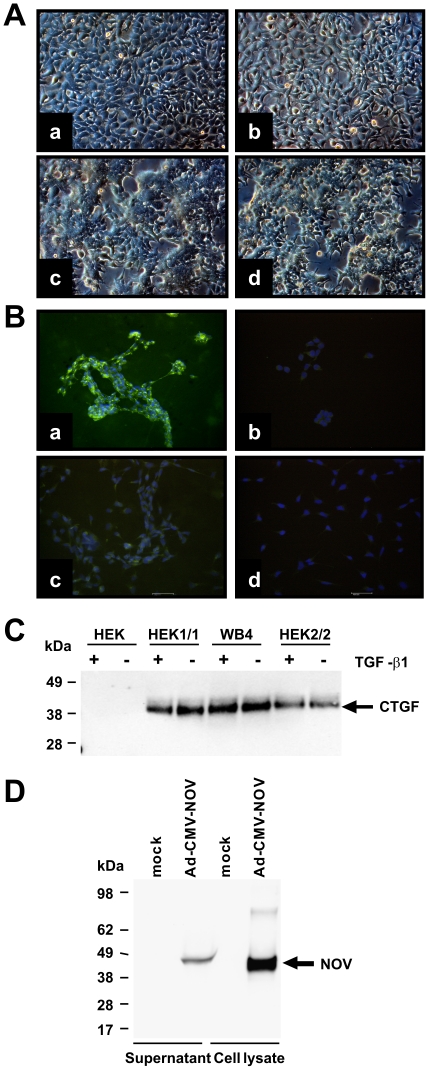
Establishing of devices for over-expressing CCN proteins. (**A**) Light microscopic images of parental HEK cells (a), Flp-In-293 (b) and stable transfected WB4 (c) and HEK1/1 (d) clones. (**B**) Immunostaining for CCN2/CTGF of stable transfected WB4 (a) and parental cells (c). Staining with an isotype control (b, d) served as control. Nuclei were counterstained with propidium iode. (**C**) CCN2/CTGF expression in stable transfected clones and parental cells was tested in Western Blot in cells stimulated with TGF-β1 or untreated cells. (**D**) Expression of CCN3/NOV was analysed in extracts and supernatants of mock- or Ad-CMV-NOV-infected COS-7 cells.

**Figure 2:**
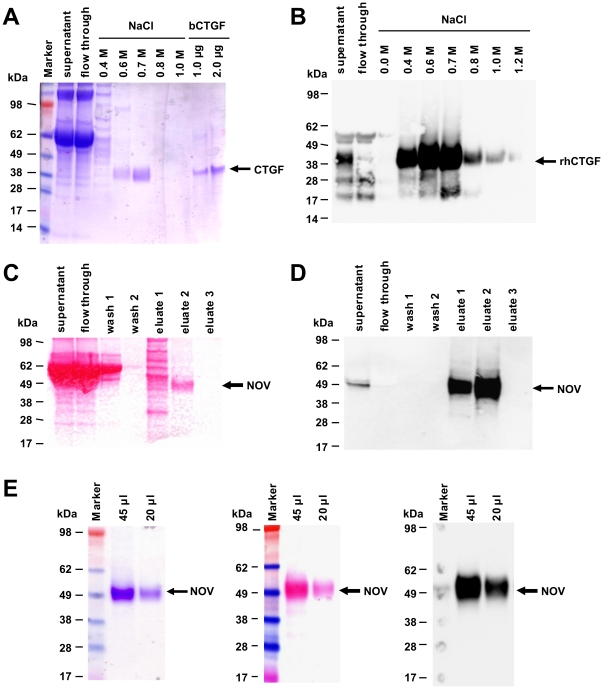
Purification of recombinant CCN proteins. (**A**) Analysis of purification of recombinant CCN2/CTGF from supernatants of stable transfected WB4 clone by SDS-PAGE and subsequent Coomassie stain. Bacterially expressed CCN2/CTGF (1 and 2 µg) served as an internal control. (**B**) Western blot analysis of individual fractions using a CCN2/CTGF-specific antibody. (**C**) Analysis of purification of CCN3/NOV from supernatants of COS-7 that were transiently infected with Ad-CMV-CCN3/NOV in Ponceau S stain. (**D**) The membrane from (**C**) was probed with a CCN3/NOV-specific antibody. (**E**) Different amounts (20 and 45 µg) of purified CCN3/NOV taken from fraction 2 (**C, D**) were analysed in Coomassie stain (*left panel*), Ponceau S stain (*middle panel*) and Western blot (*right panel*) with a CCN3/NOV-specific antibody.

**Figure 3:**
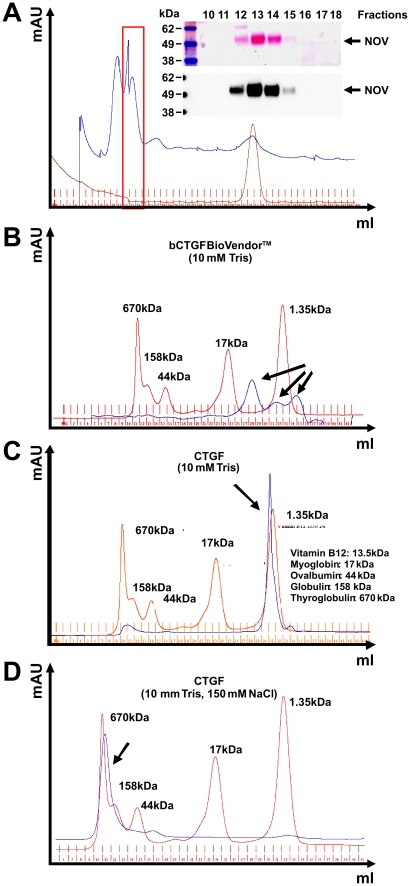
Size-exclusion chromatography of purified CCN proteins. (**A**) Size-exclusion gel chromatography of fraction 2 (cf. Figures 2C and 2D) and subsequent detection of CCN3/NOV in Western Blot. (**B**) Size-exclusion gel chromatography of bacterial expressed recombinant CCN2/CTGF (BioVendor) in a buffer containing 10 mM Tris (pH 7.0). (**C**) Size-exclusion gel chromatography of purified recombinant human CCN2/CTGF in a buffer containing 10 mM Tris (pH 7.0). (**D**) Size-exclusion gel chromatography of purified recombinant human CCN2/CTGF in a buffer containing 10 mM Tris (pH 7.0) and 0.15 M NaCl. As gel filtration standard in (**B–D**) a mixture of molecular weight markers containing Vitamin B_12_ (1.35 kDa), horse Myoglobin (17 kDa), chicken Ovalbumin (44 kDa), bovine γ-Globulin (158 kDa), and bovine Thyroglobulin (670 kDa) was applied.

We cloned expression constructs that direct the expression of full-length human CCN2/CTGF and rat CCN3/NOV with short fusions at their carboxyl-terminal regions (Figure S1). For over-expression of human CCN2/CTGF, we established stable transfected HEK293A (HEK 1/1, HEK2/2) and Flp-In-293 cell clones (WB4) that show a strong expression of respective protein (Figure 1A–1C) that is observable even after 100 cell passages (Figure S2). In addition, we established an adenoviral expression vector that directs high expression of rat CCN3/NOV (Figure 1D). Based on their property to bind heparin, we purified respective CCN proteins by use of affinity chromatography (Figure 2). Protein quantification was done with a standard protein quantification assay and specific measurement using in-house-CCN2/CTGF [35] and in-house CCN3/NOV ELISAs (Borkham-Kamphorst and Weiskirchen, unpublished) as well as Western blotting. In addition, the purity of respective recombinant CCN proteins was analysed by applying 1 µg of recombinant proteins to 4–12% Bis/Tris SDS-PAGE electrophoresis following Coomassie stain. In this analysis, recombinant human CCN2/CTGF typically appeared as two distinct bands in the range of 38–40 kDa, while rat CCN3/NOV appeared as one broad smeary band with a molecular weight of ∼48 kDa. When necessary, we further purified the proteins by Sepharose size-exclusion chromatography (Figure 3). Interestingly, the buffer salt content showed a strong influence on the elution profile of recombinant CCN proteins. Affinity purified CCN3/NOV was fractioned with a bipartite chromatogram when the gel filtration was run in a buffer that simply contained 10 mM Tris (Figure 3A). While commercially available CCN2/CTGF that was expressed in bacteria showed a tripartite elution profile (Figure 3B), the protein that we have purified from cell supernatants showed a distinct peak at very low molecular weight (Figure 3C). However, when the same protein was run in the same buffer with addition of 150 mM NaCl, we found that the peak was shifted to a range at which high molecular weight proteins were eluted (Figure 3D).

**Figure 4:**
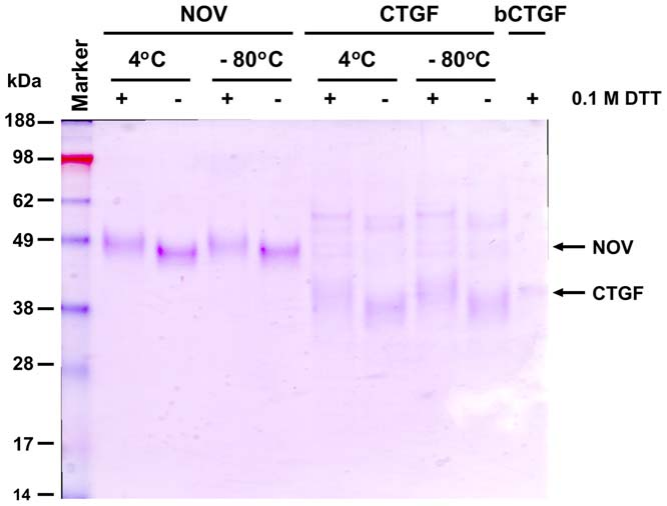
Stability of purified recombinant CCN2/CTGF and CCN3/NOV. 1 µg of purified CCN2/CTGF and CCN3/NOV that were kept for three months at indicated temperatures (4°C, −80°C) were separated on a NuPAGE Bis/Tris 4–12% gradient SDS gel under reducing and non-reducing conditions and stained with Coomassie. In this analysis 1 µg of rhCTGF (BioVendor) that was stored at −80°C served as an internal control.

**Figure 5:**
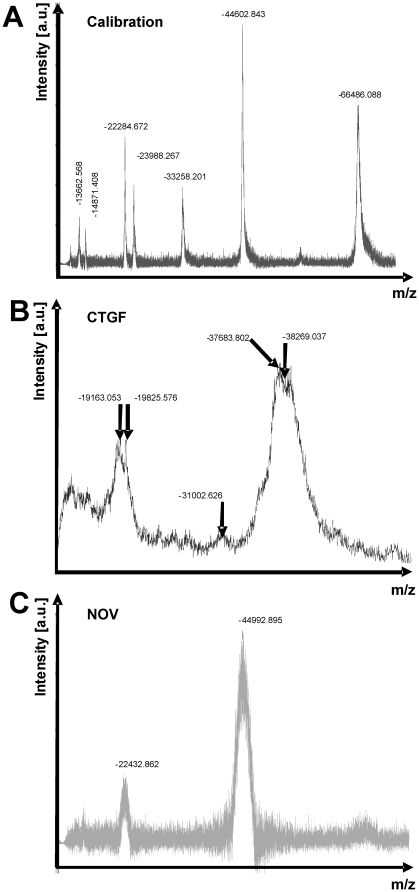
MALDI-TOF analysis of purified recombinant CCN proteins. (**A**) Calibration spectrum of MALDI-TOF mass spectroscopy using a mixture of proteins that contains Trypsinogen, Protein A, and Albumin with average m/z 23982, 44613, and ∼66500 as standards. (**B**) Mass spectrum of purified recombinant CCN2/CTGF. (**C**) Mass spectrum of purified recombinant CCN3/NOV.

**Figure 6:**
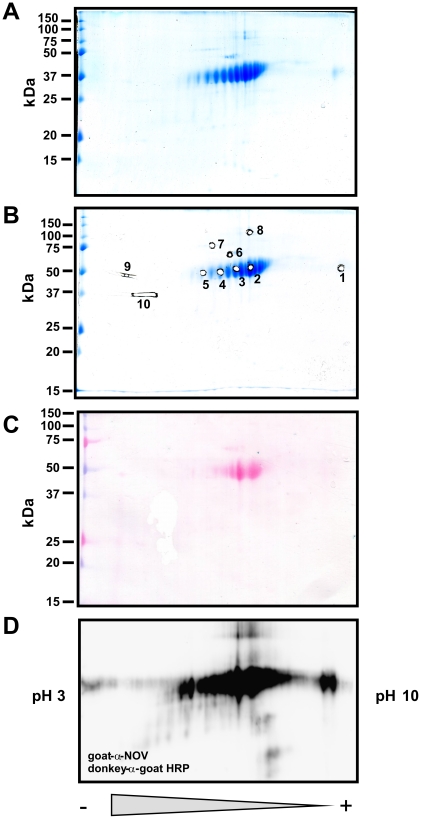
2D-SDS-PAGE, trypsin in-gel digest, ESI-TOF/MS and sequence identification of protein spots from recombinant CCN3/NOV. (**A**) 2D-SDS-PAGE of purified recombinant CCN3/NOV. The protein was first separated in its native state by isoelectric focussing in a pH gradient ranging from 3 to 10 and later by their protein mass in a denaturing SDS-PAGE. The final gel was stained with Coomassie Brilliant Blue. (**B**) Indicated spots (spot 1 to 10) were subjected to trypsin in gel digest. (**C**) A parallel gel of (**A**) was prepared and transferred without prior Coomassie stain to a Protran membrane. The membrane was stained with Ponceau S. (**D**) The membrane (**C**) was then probed with a CCN3/NOV-specific antibody.

We next tested the stability of purified recombinant CCN3/NOV and CCN2/CTGF. Therefore, we analysed respective protein solutions that were stored at 4°C and −80°C for up to three months by SDS-PAGE and Coomassie stain under reducing and non-reducing conditions (Figure 4). This analysis revealed that both proteins were rather stable and showed no significant degradation after storage. When determined the total mass of purified human CCN2/CTGF and rat CCN3/NOV by MALDI-TOF, we observed average m/z values (CCN2/CTGF, 38269 Da; CCN3/NOV, 44993 Da) that were roughly in the range of our expectation (Figure 5). However, compared to our calibration proteins (Trypsinogen, Protein A, Albumin) that produced narrow peaks (Figure 5A), the recombinant proteins produced broad peaks (Figure 5B, and 5C) in this analysis that were not really appropriate to determine the mass. To characterise this phenomenon in more detail, we next performed 2D-gelelectrophoresis in which the proteins were separated on IPG strips according to their isoelectric points (1D) and subsequently by their size using 10% SDS-PAGE (2D). Again, the recombinant proteins produced smears in these 2D gels (Figure 6) that were completely immunoreactive to specific antibodies suggesting that the complete smears resulted from different biochemical subspecies. We isolated spots/bands from 1D- (CCN2/CTGF) or 2D-gels (CCN3/NOV), subjected them to trypsin in-gel digesting and identified resulting peptides by ESI-TOF/MS and subsequent analysis using the MASCOT search engine (e.g. Table S1 and Table S2) for rapid protein identification using mass spectrometry data [37]. In this analysis, the sequence coverage was 34% for human CCN2/CTGF and 26% for rat CCN3/NOV.

**Figure 7:**
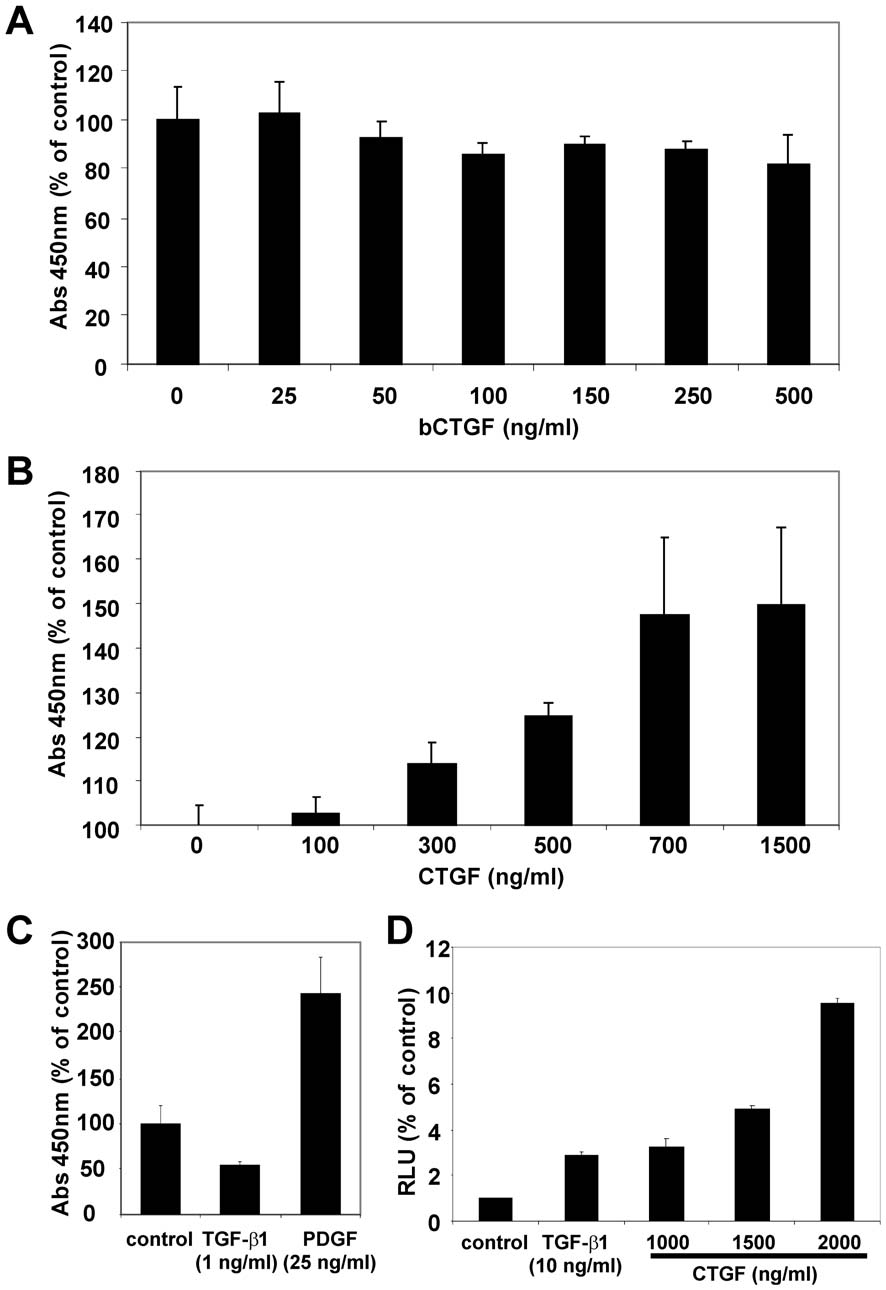
Biological activity of purified recombinant CCN2/CTGF, part I. (**A**) EA•hy 926 cells in serum-free growth medium were incubated with indicated concentrations of bacterial expressed recombinant CCN2/CTGF (BioVendor) and subjected to BrdU incorporation assay. (**B**) EA•hy 926 cells in serum-free growth medium were subjected to indicated concentration of purified recombinant CCN2/CTGF and analysed in BrdU incorporation assay. The observed proliferation rates were set in relation to control cells that received no recombinant CCN2/CTGF. (**C**) EA•hy 926 cells in serum-free growth medium were left untreated or stimulated with TGF-β1 (1 ng/ml) and PDGF-BB (25 ng/ml) and the proliferation was measured in BrdU assay. (**D**) EA•hy 926 cells were transiently transfected with the (CAGA)_12_-Luc reporter plasmid and incubated with TGF-β1 (10 ng/ml), indicated concentrations of recombinant CCN2/CTGF or left unstimulated. The relative luciferase activity was measured after 24 hr. The gene promoter activity of the unstimulated control cells was set to 1 in this experiment.

Subsequently, we demonstrated the biological activity of the purified proteins. The biological activity of recombinant CCN2/CTGF was proven by stimulation of endothelial cell line EA•hy 926 that represents a human intraspecies hybrid [33] that was previously tested to be highly sensitive against CCN2/CTGF [34]. When cells were stimulated for 24 h with different concentrations of purified CCN2/CTGF (100–2000 ng/ml) in serum-free medium, we observed a dosis-dependent increase of proliferation, while bacterial expressed CCN2/CTGF (bCTGF) that is commercially showed no stimulatory activity in this proliferation assay (Figure 7). Recombinant CCN2/CTGF induced a 1.6 fold BrdU incorporation compared to unstimulated controls. PDGF-BB (25 ng/ml) and TGF-β1 (1 ng/ml) that were incorporated in this assay as positive and negative controls induced a 2.9-fold induction or a 50% suppression of BrdU incorporation. Purified rat CCN3/NOV showed no effect on the BrdU incorporation in this assay (data not shown).

**Figure 8:**
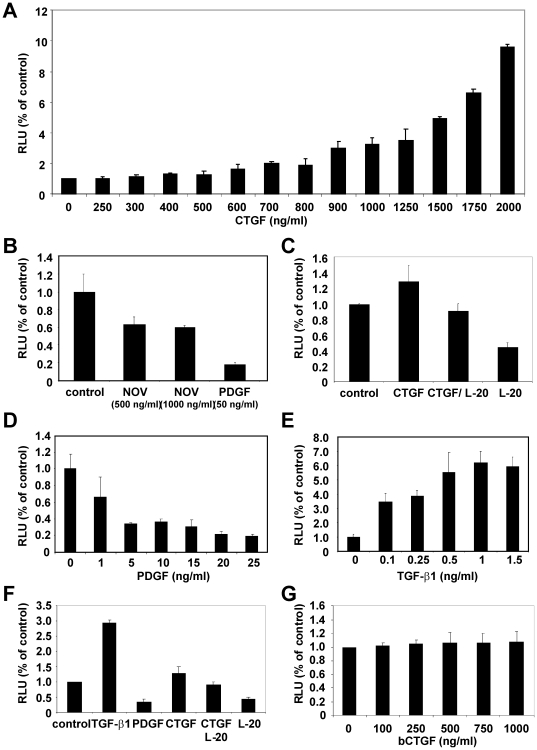
Biological activity of purified recombinant CTGF, part II. (**A**) EA•hy 926 cells were transfected with the (CAGA)_12_-Luc reporter plasmid and incubated with indicated concentrations of recombinant human CCN2/CTGF. Cell lysates were prepared after 24 hr and luciferase activity determined. In this assay, the luciferase activity of normal control cells (0 ng/ml) was set to 1. The experiments were repeated three times. Shown are the means ±SD of one representative experiment performed in triplicate. (**B**) EA•hy 926 cells were transfected with the reporter plasmid and incubated with different concentrations of CCN3/NOV (500 or 1000 ng/ml) or PDGF (50 ng/ml) in serum-free medium. The promoter activity of the unstimulated controls was set as 1. The experiment was done three times in triplicate. The diagram depicts the means ± SD of one representative experiment. (**C**) After transfection with the Smad3 sensitive reporter, EA•hy 926 cells were incubated with recombinant CCN3/CTGF, a combination of CCN2/CTGF and CCN2/CTGF-specific antibody L-20, L-20 alone, or left untreated. 24 hr later, the relative luciferase activity was determined. The experiment was repeated three times. Shown are the results (means ±SD) of one representative experiment that was performed in triplicate. (**D–G**) EA•hy 926 cells were transiently transfected with the (CAGA)_12_-Luc reporter plasmid and incubated with indicated concentrations/combinations of PDGF-BB, TGF-β1, CCN2/CTGF, CCN2/CTGF capturing antibody (L-20, sc-14939), and bacterial expressed CCN2/CTGF in serum-free medium. The promoter activity was measured 24 hr later.

To analyse if CCN2/CTGF interferes with TGF-β signalling, we performed reporter gene assays in with EA•hy 926 cells using a reporter containing Smad3/Smad4 binding sequences, termed CAGA boxes, that were originally derived from the promoter of the human PAI-1 gene and is a read-out-system for phosphorylated Smad3 [32]. We transfected EA•hy 926 cells with the reporter (CAGA)_12_-MLP-Luc vector and stimulated the cells in serum-free DMEM for 24 hr with different concentrations/combinations of recombinant CCN2/CTGF, CCN3/NOV, TGF-β1, PDGF, bCTGF, and an antagonizing antibody (L-20) directed against CCN2/CTGF (Figure 8). The obtained luciferase activities were normalised to the protein amount and calculated as light count per second per µg protein (lcps/µg protein) and relative luciferase units (RLU) were compared to un-stimulated controls that were set to 1. In this assay, TGF-β1 (1 ng/ml) stimulation leads to a 6.2-fold increase, while PDGF-BB (50 ng/ml) stimulation decreases luciferase activity to 0.18 of the unstimulated control. Stimulation with 2 µg/ml of purified CCN2/CTGF induced the luciferase activity by a factor of 10, while the stimulation with 1 µg/ml of purified NOV decreased the luciferase activity to 0.6 fold. In the presence of the capturing antibody, the stimulatory effect of CCN2/CTGF on luciferase activity was negotiated. In line, the capturing antibody itself decreases luciferase activity to 0.4-fold of unstimulated control suggesting that the biological activity of endogenous expression of CCN2/CTGF in EA•hy 926 cells (Figure S3) is blocked by sequestering. There was no increase of luciferase activity observable in the bCTGF-stimulated and transiently transfected EA•hy 926 cells. We could show a significant difference between stimulated (500 ng/ml, 1000 ng/ml) and unstimulated EA•hy 926 cells (p≤0.05) using double-sided t-test (Figure S4).

**Figure 9:**
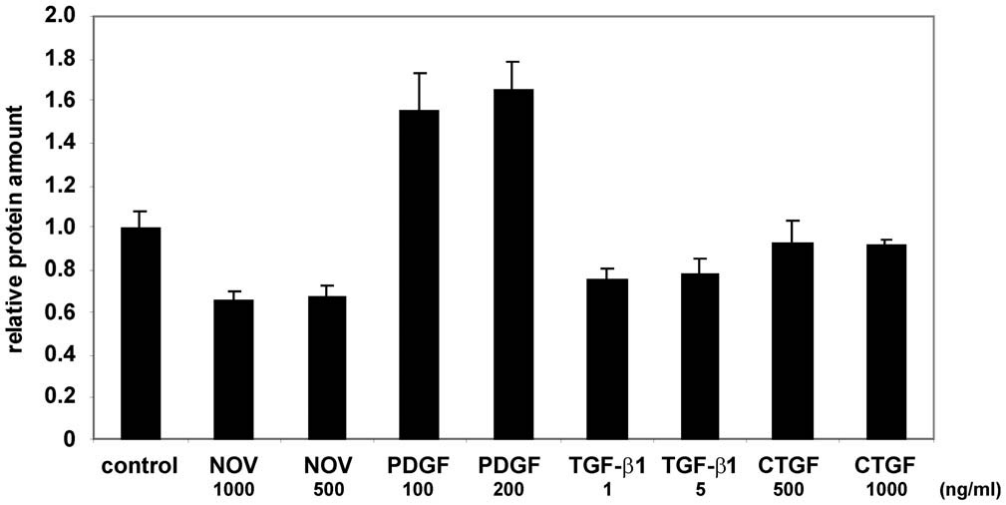
Glycosylation of CCN proteins. (**A–D**) Purified fraction of CCN3/NOV (**A, B**) or CCN2/CTGF (**C, D**) that were stored at indicated temperatures for three months were subjected to denaturing (+ DTT) and non-denaturing (− DTT) SDS-PAGE (Bis/Tris 4–12% gradient). The gels were blotted onto Protran membranes and subsequently probed with a CCN3/NOV- (**A**), a CCN2/CTGF- (**C**), or a ConA-HRP conjugate (**B, D**). In this analysis, a soluble PDGF type β receptor (PDGFRβ) was used as a highly glycosylated protein control. BSA and the bacterial expressed CCN3/CTGF (BioVendor) were used as negative controls. In this set of experiments the blots A and C were cut at indicated cut edges prior incubation with antibodies directed against CCN proteins or PDGFRβ.

We next tested the effect of the purified proteins on biochemical cell activity. Therefore, equal cell numbers of EA•hy 926 cells were seeded, serum-starved overnight and stimulated with TGF-β1, PDGF-BB, recombinant human CCN2/CTGF and rat CCN3/NOV for 24 hr. After the incubation period, the cells were washed and whole cell lysates for comparative quantification (the amount of proteins in unstimulated cells were set to 1) of total protein concentration prepared. We found that cells treated with PDGF-BB had a 1.6-fold higher protein amount than unstimulated controls. In EA•hy 926 cells that were stimulated with CCN3/NOV (500 ng/ml), the protein amount was decreased to 0.6-fold of the unstimulated control. Likewise, stimulation with 1 ng/ml of TGF-β1 decreased the protein amount to 0.75-fold of the unstimulated control. CCN2/CTGF had no influence on the protein amount (Figure 9).

**Figure 10:**
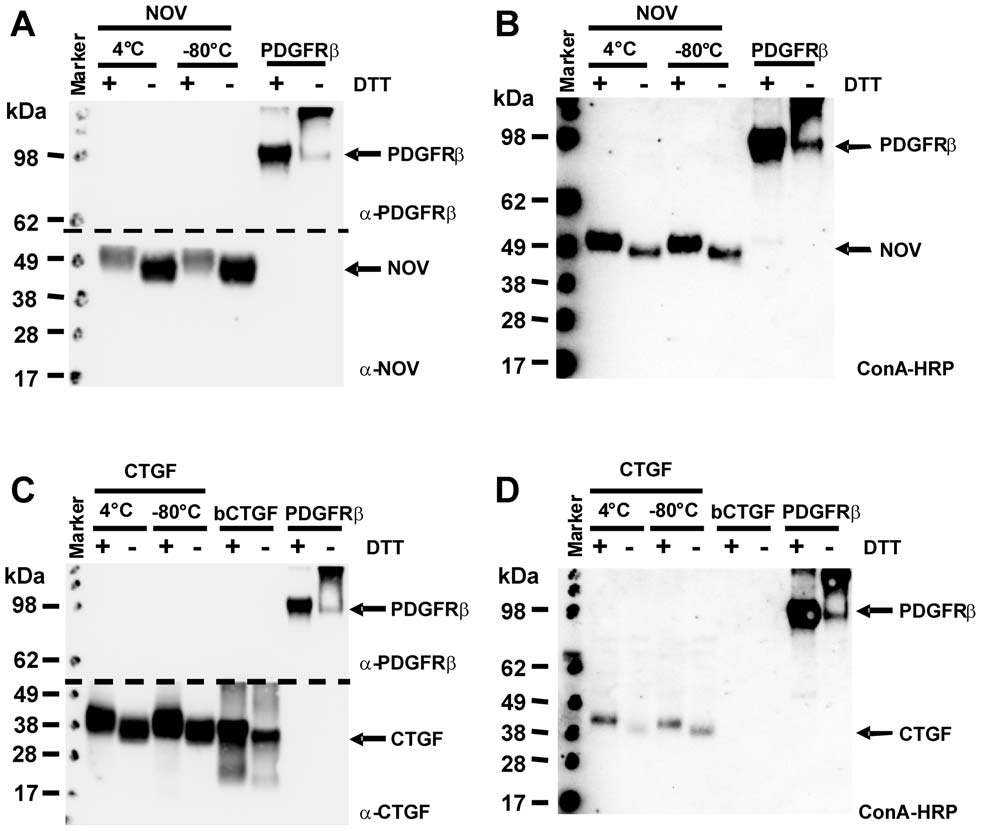
Deglycosylation of CCN proteins. (**A**) 1 µg of recombinant CCN3/NOV or CCN2/CTGF was subjected to deglycosylation assay using the endoglycosidase Endo H (removes only high mannose and some hybrid types of N-linked carbohydrates) and PNGase F (removes carbohydrates attached to an Asn). The digested proteins were then resolved by SDS-PAGE (NuPAGE Bis/Tris 4–12% gradient) and probed with indicated antibodies.

**Figure 11:**
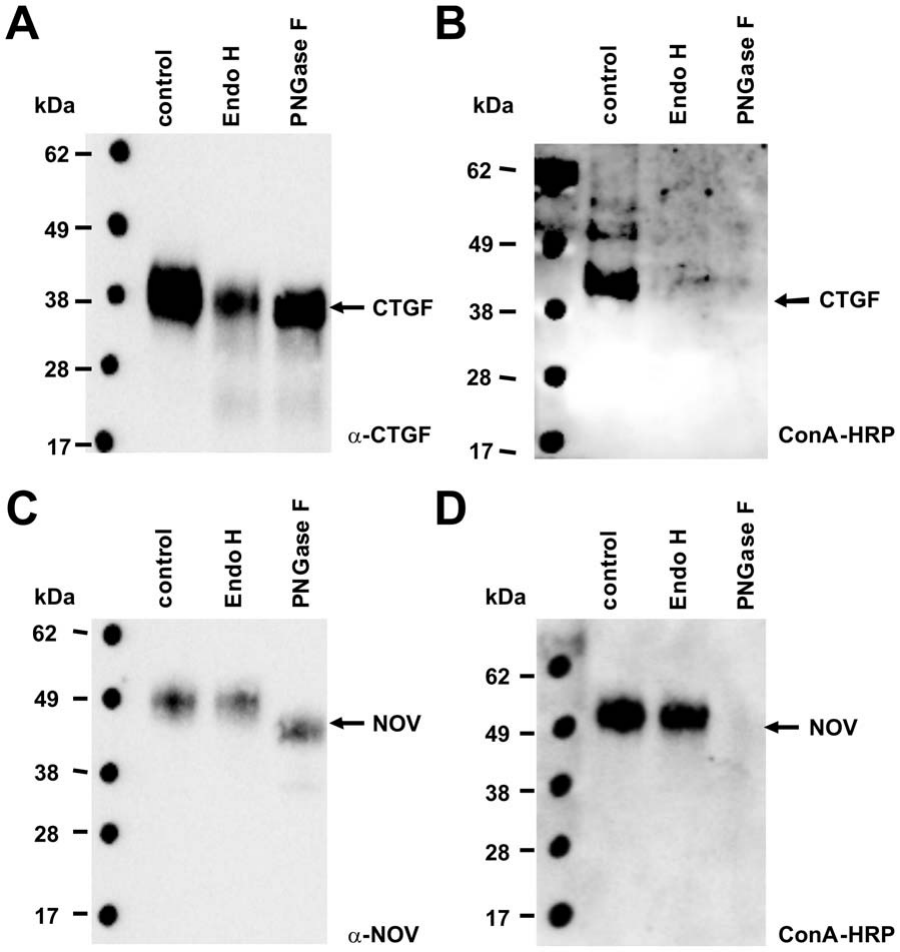
Measurement of mitogenic activity of different growth factors. EA•hy 926 cells were incubated with different concentrations of growth factors in serum-free medium. Protein amounts of whole cell lysates were measured by Micro BCA assay after 48 hr. In this analysis, the protein amount in untreated cells was set to 1. The experiments were repeated three times. Shown are the means ± SD of one representative experiment that was performed in triplicate.

Based on the finding that the purified CCN proteins appeared not as unique populations in gel electrophoresis (cf. Figure 6) and showed atypical chromatography behaviour (cf. Figure 3), we analysed if the heterogeneity resulted from glycosylation. When we analysed the human CCN2/CTGF and rat CCN3/NOV protein sequences for potential N-glycosylation sites using the NetNGlyc algorithm (http://www.cbs.dtu.dk/services/NetNGlyc/), we found that both proteins carry a potential N-glycosylation site (Figure S5). To test if the proteins are indeed N-glycosylated, we first analysed if the respective proteins have lectin binding capacity using a Con A-HRP conjugate (Figure 10). As negative and positive controls, 1 µg BSA and the soluble highly glycosylated extracellular part of the PDGF-BB receptor [38] was incorporated into this assay. Additionally, bacterial expressed recombinant CTGF (bCTGF) that should contain no sugar groups was analysed in parallel. In this analysis, we found that both recombinant proteins from eukaryotic sources and the extracellular domain from the PDGF type β receptor (PDGFRβ) were detectable with the Concanavalin (ConA)-HRP conjugate suggesting that these proteins carry sugar groups. In contrast, CCN2/CTGF that originated from a bacterial source was only detected by the L-20 antibody but not by Con A. In a second set of experiments, we tried if the purified CCN proteins could be enzymatically deglycosylated by Endo H (removes only high mannose and some hybrid types of N-linked carbohydrates) or PNGase F (removes all types of N-linked carbohydrates). This analysis revealed that incubation of CTGF with one of both enzymes was suitable to remove the affinity for the Con A-HRP conjugate, while the respective affinity of CCN3/NOV was only removed after treatment with PNGase F (Figure 11). In addition, the treatment with PNGase F resulted in a marked alteration of electrophoretic mobility.

**Figure 12:**
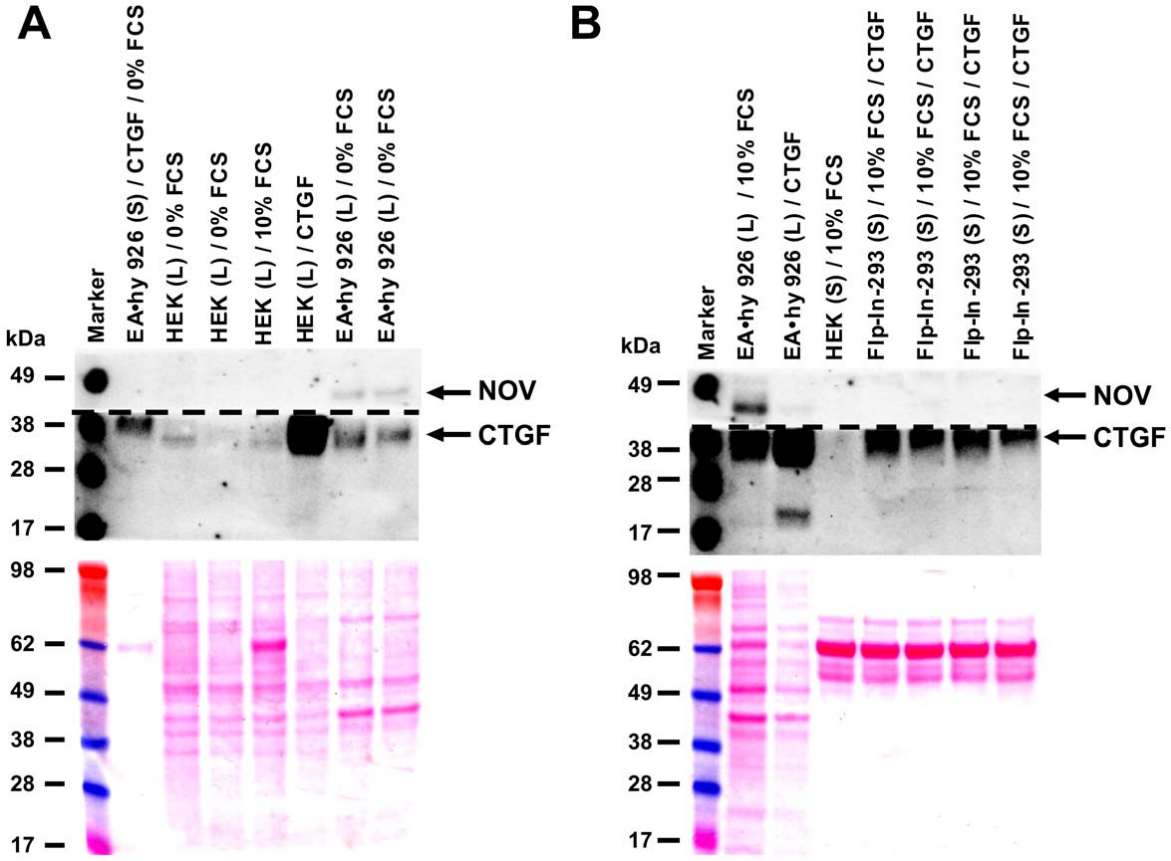
Yin and Yang regulation of CCN3/NOV and CCN2/CTGF. (**A**) Supernatants (*S*) or protein cell extracts (*L*) of EA•hy 926 cells or parental HEK cells that were incubated under indicated conditions (24 hr) were analysed for expression of CCN3/NOV and CCN2/CTGF in Western blot. (**B**) EA•hy 926 cells were cultured in the presence of conditioned media taken from Flp-In-293 clones over-expressing CCN2/CTGF. Cell lysates (*L*) or supernatants (*S*) were then tested for expression of CCN3/NOV and CCN2/CTGF by Western blot. Shown are representatives of two independent experiments.

A previous study reports that CCN2/CTGF and CCN3/NOV can regulate their expression in an Yin/Yang manner and that either the addition or over-expression of CCN3/NOV produces a marked down-regulation of CCN2/CTGF in mesangial cells [25]. To test if this effect is also reproducible in EA•hy 926 cells, equal cell numbers were seeded, serum-starved overnight, and cultured in conditioned medium enriched in recombinant human CCN2/CTGF. Thereafter, the cells were washed, whole cell lysates prepared and analysed for CCN3/NOV expression revealing that EA•hy 926 cells, that normally express both CCN proteins, produce less amounts of endogenous CCN3/NOV after CCN2/CTGF treatment (Figure 12).

## Discussion

Protein studies aiming to unravel biological functions require pure and native proteins that might be further dependent on their correct post translational modifications. It is known that the correct configuration of carbohydrate chains is important in the signalling of growth factors [39].

In this study, we have established novel devices for expression of recombinant human CCN2/CTGF and rat CCN3/NOV (Figure 1). By use of heparin affinity and size exclusion chromatography, we were able to purify both CCN proteins to heterogeneity (Figure 2). Typically, these expression devices allow to purify 200–300 µg of recombinant human CCN2/CTGF from 400 ml growth medium of stable transfected Flp-In-293 clones and approximately 300 µg recombinant rat CCN3/NOV from 400 ml conditioned supernatant of COS-7 cells that were infected with Ad5-CMV-rNOV. The identity of purified proteins was demonstrated by mass spectrometry (Figure 5) and trypsin in-gel-digest (Figure 6) following identification of resulting peptides by ESI-MS/MS and subsequent analysis using the MASCOT algorithm.

We further demonstrate that the purified proteins are highly stable when stored at −80°C or 4°C (Figure 4). Interestingly, the retention time of purified CTGF in size exclusion chromatography was strongly dependent on the salt content of chosen buffer. Low salt concentration prolonged the retention time, while higher salt concentrations (150 mM) leads to shorter retention times (Figure 3). Although this phenomenon is known for many other proteins, the drastic shift of the elution time might indicate that CCN2/CTGF strongly interacts with the sepharose matrix representing a crosslinked, beaded-form of a polysaccharide polymer material (agarose) that is crosslinked through lysine side chains.

The biological activity of the isolated proteins is the key indicator of successful expression. For recombinant CCN2/CTGF we have shown the biological activity by use of its positive influence on proliferation in EA•hy 926 cells [34] and in a reporter gene assays using the Smad3/4-sensitve reporter (i.e. (CAGA)_12_-MLP-Luc)) that originally originated from the human PAI-1 gene promoter [32]. After addition of CCN2/CTGF, we found an increase of proliferation in BrdU incorporation assay of around 150% (Figure 7), values that were similar to those observed in pancreatic stellate cells [40]. In this assay, the EA•hy 926 cells were also increased after stimulation with PDGF-BB and decreased after TGF-β1 stimulation and moreover bacterial expressed CCN2/CTGF failed to influence proliferation of EA•hy 926 cells. The results obtained after transient transfection of EA•hy 926 cells with the reporter vector (CAGA)_12_-MLP-Luc showed an increase of the firefly luciferase read that was dose-dependent and blocked by the addition of an antagonistic antibody (Figure 8). Even more, this antibody was suitable to block the stimulatory effect of endogenous CCN2/CTGF. Contrarily, the stimulation with recombinant NOV leads to a 0.6-fold decrease in luciferase activity suggesting that NOV might evolve opposing activities towards CCN2/CTGF. That finding fits well with the recent hypothesis that the expression of CCN2/CTGF and CCN3/NOV is controlled in an Yin/Yang manner [25] and was further highlighted by the fact that the expression of CCN3/NOV in EA•hy 926 cells could be decreased by supplementation of growth medium with CTGF (Figure 12).

The most interesting finding of this study is the demonstration that both purified recombinant CCN proteins are N-glycosylated (Figures 10 and 11). Both proteins (i) have affinity for the Con A-HRP conjugate that is lost after treatment with endoglycosidases, (ii) migrate as diffuse bands in SDS-PAGE, and (iii) appear not uniform in mass spectrometry. We could demonstrate a prominent shift in molecular weight after deglycosylation with PNGase F for CCN3/NOV in SDS gel electrophoresis that is associated with a reduced lectin binding activity. Likewise CCN2/CTGF that originated from eukaryotic sources had affinity for the Con A-conjugate that was not found in CCN2/CTGF that was expressed in bacteria and lost after treatment with either PNGase F or Endo H (Figure 11). Prediction that have been done on the ExPASy Proteomics Server show two N-glycosylation sites at positions 28 NCSG (high potential) and 225 NASC (low potential) in human CCN2/CTGF and at positions 91 NETG (potential) and 274 NCTS (low potential) in rat NOV. There were no predictions for O-glycosylation sites in human CCN2/CTGF and only one predicted O-glycosylation site at threonine 43. The activity of growth factors and signalling proteins are depended on the post-translational modifications including glycosylation and correct building of the intramolecular disulfide bond network. These modifications are difficult to perform in *E. coli* expressing system [41] but obviously accurately done in our eukaryotic expression systems (i.e. HEK, COS-7). Although we do not yet know if N-glycosylation is a post-translational modification that is important for the folding or biological activity of these CCN proteins, it might be an essential prerequisite for proper protein secretion and adhesion/incorporation into the ECM or to prevent premature degradation.

Incubation of EA•hy 926 cells with conditioned medium isolated from WB4 over-expressing human CCN2/CTGF resulted in an increase of endogenous CTGF and suppression of endogenous CCN3/NOV. This data confirms previous suggestions that exogenous CCN2/CTGF is able to suppress expression of CCN3/NOV in a Yin/Yang manner [25].

In summary, we have established an expression and purification system to manufacture biological active recombinant human CCN2/CTGF and rat CCN3/NOV in permanent mammalian cell lines. Both CCN proteins are rather stable and can be stored at 4°C or −80°C for at least 3 month without disintegration. The biological activity of both proteins was demonstrated in gene reporter and proliferation assays.

The opportunity to prepare large quantities of biological active CCN2/CTGF and CCN3/NOV proteins will now allow us to address key questions of these CCN proteins in regard to structure, function, post-translational modifications (e. g. glycosylation), involvement in individual signal cascades, and further provide a foundation to address their therapeutic potential in various disease models.

## Supporting Information

Figure S1 **Recombinant CCN proteins expressed in this study.** (**A, B**) Sequence of modified human CCN2/CTGF (**A**) and rat CCN3/NOV (**B**) expressed in this study. The predicted leader sequences are depicted in blue and the amino acid introduced by the chosen cloning strategy in red. The numbers of amino acid positions are depicted on the left margin. Potential cleavage sites are marked by arrows. In addition the theoretical isoelectric points (pI) and molecular weights (Mw) of expressed proteins, endogenous proteins, and expressed fusion proteins after removal of leader sequence are given in red, black and blue, respectively.
(TIF)

Figure S2 **CCN2/CTGF expression in selected clones after prolonged passages.** The expression of CCN2/CTGF in cell extracts (*L*) and supernatants (*S*) of indicated clones that were cultured in medium containing different amounts of FCS was analyzed by Western blot. In this analysis purified recombinant CCN2/CTGF was used as a positive control.
(TIF)

Figure S3 **Analysis of CCN2/CTGF expression in different cell lines that were used in this study.** A Western blot analysis was performed to analyze CCN2/CTGF expression in cell extracts of indicated cell lines that were cultured in medium containing different amounts of FCS. Purified recombinant CCN2/CTGF was used as a positive control.
(TIF)

Figure S4 **Statistical analysis of reporter gene assays.** The graph depicts the results of a two-sided t-test of three individual (CAGA)_12_-MLP-Luc reporter gene assays in which EA•hy 926 cells were stimulated with indicated proteins/cytokines.
(TIF)

Figure S5 **Potential N-glycosylation sites.** A NetNGlyc-analysis was performed with (**A**) human CCN2/CTGF and (**B**) rat CCN3/NOV. Both proteins contain two potential N-glycosylation sites at indicated positions.
(TIF)

Table S1 **Identification of fragments by ESI-TOF/MS for human CCN2/CTGF.**
(DOC)

Table S2 **Identification of fragments by ESI-TOF/MS for rat CCN3/NOV.**
(DOC)
