# Supplementary figures and images for: Recombinant Expression, Purification, and Functional Characterisation of Connective Tissue Growth Factor and Nephroblastoma-Overexpressed Protein
Source: PLoS One. 2010 Dec 30;5(12):e16000. doi: 10.1371/journal.pone.0016000 (PMC3012735; doi:10.1371/journal.pone.0016000)

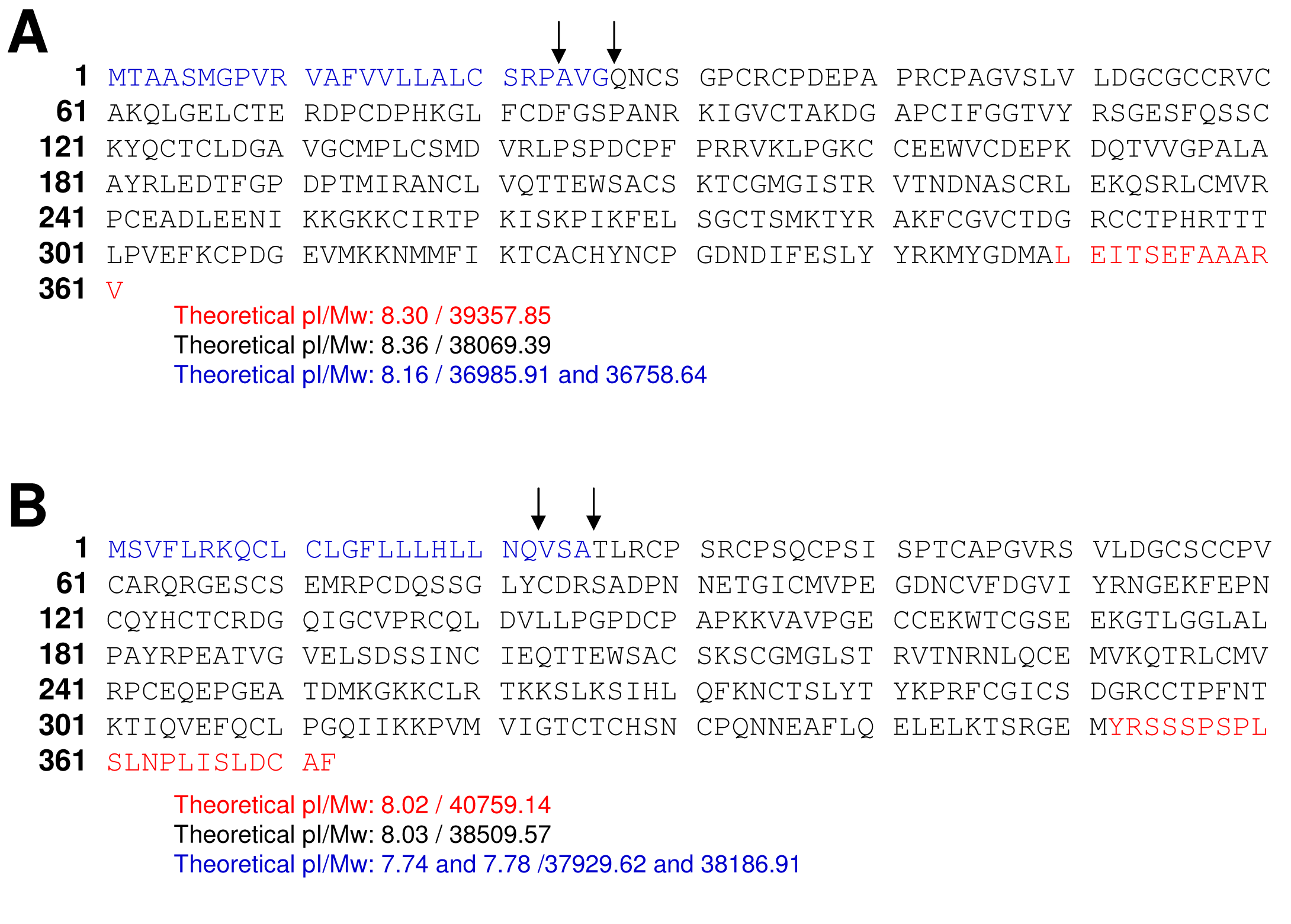

Supplement: Figure S1 — Recombinant CCN proteins expressed in this study. (A, B) Sequence of modified human CCN2/CTGF (A) and rat CCN3/NOV (B) expressed in this study. The predicted leader sequences are depicted in blue and the amino acid introduced by the chosen cloning strategy in red. The numbers of amino acid positions are depicted on the left margin. Potential cleavage sites are marked by arrows. In addition the theoretical isoelectric points (pI) and molecular weights (Mw) of expressed proteins, endogenous proteins, and expressed fusion proteins after removal of leader sequence are given in red, black and blue, respectively. (TIF) [file pone.0016000.s001.tif]
