# Supplementary material for: Recombinant Expression, Purification, and Functional Characterisation of Connective Tissue Growth Factor and Nephroblastoma-Overexpressed Protein
Source: PLoS One. 2010 Dec 30;5(12):e16000. doi: 10.1371/journal.pone.0016000 (PMC3012735; doi:10.1371/journal.pone.0016000)
Supplement: Table S1 — Identification of fragments by ESI-TOF/MS for human CCN2/CTGF. (DOC) [file pone.0016000.s006.doc]

**Supplementary Table 1**

**Identification of fragments by ESI-TOF/MS for human CCN2/CTGF***

| **Spot ID** | **Number of individual peptide peaks analysed** | **Identified sequences** |
| --- | --- | --- |
| **38 kDa** | **7** | **R.TTTLPVEFK.C** |
|  |  | **R.LPSPDCPFPR.R** |
|  |  | **K.DQTVVGPALAAYR.L** |
|  |  | **K.DGAPCVFGGTVYR.S** |
|  |  | **R.LEDTFGPDPTMIR.A** |
|  |  | **R.LEDTFGPDPTMLR.A** |
|  |  | **K.DRTAVGPALAAYR.L** |
| **1** MTAASMGPVRVAFVVLLALCSRPAVGQNCSGPCRCPDEPAPRCPAGVSLVLDGCGCCRVC  **61** AKQLGELCTERDPCDPHKGLFCDFGSPANRKIGVCTAKDGAPCIFGGTVYRSGESFQSSC  **121** KYQCTCLDGAVGCMPLCSMDVRLPSPDCPFPRRVKLPGKCCEEWVCDEPKDQTVVGPALA  **181** AYRLEDTFGPDPTMIRANCLVQTTEWSACSKTCGMGISTRVTNDNASCRLEKQSRLCMVR  **241** PCEADLEENIKKGKKCIRTPKISKPIKFELSGCTSMKTYRAKFCGVCTDGRCCTPHRTTT  **301** LPVEFKCPDGEVMKKNMMFIKTCACHYNCPGDNDIFESLYYRKMYGDMALEITSEFAAAR  **361** V | | |

*** Red:** Trypsin cleavage sites (R or K); **Blue:** identified sequence stretch; **Pink:** mis-interpreted amino acid because of marginal mass differences
