# Supplementary material for: Recombinant Expression, Purification, and Functional Characterisation of Connective Tissue Growth Factor and Nephroblastoma-Overexpressed Protein
Source: PLoS One. 2010 Dec 30;5(12):e16000. doi: 10.1371/journal.pone.0016000 (PMC3012735; doi:10.1371/journal.pone.0016000)
Supplement: Table S2 — Identification of fragments by ESI-TOF/MS for rat CCN3/NOV. (DOC) [file pone.0016000.s007.doc]

**Supplementary Table 2**

**Identification of fragments by ESI-TOF/MS for rat CCN3/NOV***

| **Spot ID** | **Number of individual peptide peaks analysed** | **Identified sequences** |
| --- | --- | --- |
| **1** | **1** | **R.CQLDVLLPGPDCPAPK.K** |
| **2** | **10** | **R.SVLDGCSCCPVCAR.Q** |
|  |  | **R.QRGESCSEMRPCDQSSGLYCDR.S** |
|  |  | **R.GESCSEMRPCDQSSGLYCDR.S** |
|  |  | **R.CQLDVLLPGPDCPAPK.K** |
|  |  | **R.LCMVRPCEQEPGEATDMK.G** |
|  |  | **K.SIHLQFK.N** |
|  |  | **K.TIQVEFQCLPGQIIK.K** |
|  |  | **R.SVLDGCSCCPVCAR.Q** |
|  |  | **K.TIQVEFQCLPGEIIK.K** |
|  |  | **R.QRGESCSEMRPCDQSSGLYCDR.S** |
|  |  | **R.GESCSEMRPCDQSSGLYCDR.S** |
| **3** | **9** | **K.SIHLQFK.N** |
|  |  | **R.SVLDGCSCCPVCAR.Q** |
|  |  | **K.TIQVEFQCLPGQIIK.K** |
|  |  | **K.TIQVEFQCLPGQIIK.K** |
|  |  | **R.CQLDVLLPGPDCPAPK.K** |
|  |  | **R.LCMVRPCEQEPGEATDMK.G** |
|  |  | **R.GESCSEMRPCDQSSGLYCDR.S** |
|  |  | **K.TIQVEFQCLPGEIIK.K** |
|  |  | **R.SVLDGCSCCPVCAR.Q** |
|  |  | **R.GESCSEMRPCDQSSGLYCDR.S** |
| **4** | **6** | **K.SIHLQFK.N** |
|  |  | **R.SVLDGCSCCPVCAR.Q** |
|  |  | **K.TIQVEFQCLPGEIIK.K** |
|  |  | **R.CPSQCPSISPTCAPGVR.S** |
|  |  | **R.LCMVRPCEQEPGEATDMK.G** |
|  |  | **R.SVLDGCSCCPVCAR.Q** |
|  |  | **R.CQLDVLLPGPDCPAPK.K** |
| **5** | **4** | **R.SVLDGCSCCPVCAR.Q** |
|  |  | **K.TIQVEFQCLPGQIIK.K** |
|  |  | **R.CQLDVLLPGPDCPAPK.K** |
|  |  | **K.TIQVEFQCLPGEIIK.K** |
| **6** |  | **no fragment of NOV** |
| **7** |  | **no fragment of NOV** |
| **8** | **2** | **K.TIQVEFQCLPGQIIK.K** |
|  |  | **R.CQLDVLLPGPDCPAPK.K** |
| **9** |  | **no fragment of NOV** |
| **10** |  | **no fragment of NOV** |
| **1** MSVFLRKQCLCLGFLLLHLLNQVSATLRCPSRCPSQCPSISPTCAPGVRSVLDGCSCCPV  **61** CARQRGESCSEMRPCDQSSGLYCDRSADPNNETGICMVPEGDNCVFDGVIYRNGEKFEPN  **121** CQYHCTCRDGQIGCVPRCQLDVLLPGPDCPAPKKVAVPGECCEKWTCGSEEKGTLGGLAL  **181** PAYRPEATVGVELSDSSINCIEQTTEWSACSKSCGMGLSTRVTNRNLQCEMVKQTRLCMV  **241** RPCEQEPGEATDMKGKKCLRTKKSLKSIHLQFKNCTSLYTYKPRFCGICSDGRCCTPFNT  **301** KTIQVEFQCLPGQIIKKPVMVIGTCTCHSNCPQNNEAFLQELELKTSRGEMYRSSSPSPL  **361** SLNPLISLDCAF | | |

*** Red:** Trypsin cleavage sites (R or K); **Blue:** identified sequence stretch
